# The Cervix Cancer Research Network: Increasing Access to Cancer Clinical Trials in Low- and Middle-Income Countries

**DOI:** 10.3389/fonc.2015.00014

**Published:** 2015-02-19

**Authors:** Gita Suneja, Monica Bacon, William Small, Sang Y. Ryu, Henry C. Kitchener, David K. Gaffney

**Affiliations:** ^1^Department of Radiation Oncology, University of Utah School of Medicine, Salt Lake City, UT, USA; ^2^GCIG: Gynecologic Cancer Intergroup, Kingston, ON, Canada; ^3^Department of Radiation Oncology, Stritch School of Medicine Loyola University, Chicago, IL, USA; ^4^Department of Surgery, Division of Gastroenterologic Surgery, Chonnam National University Medical School, Gwangju, South Korea; ^5^Institute of Cancer Sciences, St Mary’s Hospital, University of Manchester, Manchester, UK

**Keywords:** cervix cancer, global health, radiation oncology, clinical trials as topic, HIV cancer

## Abstract

**Introduction:** The burden of cervical cancer is large and growing in developing countries, due in large part to limited access to screening services and lack of human papillomavirus (HPV) vaccination. In spite of modern advances in diagnostic and therapeutic modalities, outcomes from cervical cancer have not markedly improved in recent years. Novel clinical trials are urgently needed to improve outcomes from cervical cancer worldwide.

**Methods:** The Cervix Cancer Research Network (CCRN), a subsidiary of the Gynecologic Cancer InterGroup, is a multi-national, multi-institutional consortium of physicians and scientists focused on improving cervical cancer outcomes worldwide by making cancer clinical trials available in low-, middle-, and high-income countries. Standard operating procedures for participation in CCRN include a pre-qualifying questionnaire to evaluate clinical activities and research infrastructure, followed by a site visit. Once a site is approved, they may choose to participate in one of four currently accruing clinical trials.

**Results:** To date, 13 different CCRN site visits have been performed. Of these 13 sites visited, 10 have been approved as CCRN sites including Tata Memorial Hospital, India; Bangalore, India; Trivandrum, India; Ramathibodi, Thailand; Siriaj, Thailand; Pramongkutklao, Thailand; Ho Chi Minh, Vietnam; Blokhin Russian Cancer Research Center; the Hertzen Moscow Cancer Research Institute; and the Russian Scientific Center of Roentgenoradiology. The four currently accruing clinical trials are TACO, OUTBACK, INTERLACE, and SHAPE.

**Discussion:** The CCRN has successfully enrolled eight sites in developing countries to participate in four randomized clinical trials. The primary objectives are to provide novel therapeutics to regions with the greatest need and to improve the validity and generalizability of clinical trial results by enrolling a diverse sample of patients.

## Introduction

Cervical cancer is the fourth most common cancer in women worldwide with almost 530,000 cases diagnosed in 2012 (1). Of these, nearly 85% of cases occur in developing countries, due in large part to limited access to screening services and lack of human papillomavirus (HPV) vaccination (2). Lack of screening also leads to diagnosis at advanced stages of disease and with clinically debilitating symptoms. Even more concerning, nearly 90% of the estimated 270,000 deaths from cervical cancer in 2012 occurred in developing countries (1), suggesting that cancer diagnosis and treatment services are inadequate in regions of the world with the highest incidence of the disease. Furthermore, cervical cancer disproportionately affects young women, and loss of life attributable to advanced cancer has significant social and economic impact on individual families, as well as societies at large (3–5).

Treatment for cervical cancer can include surgery, chemotherapy, radiotherapy, or a combination of these treatments depending on the stage at cancer diagnosis (6). For locally advanced disease, concurrent chemoradiation followed by brachytherapy has been the standard of care in developed nations for decades based on the results of several large, randomized clinical trials showing improvement in survival with the addition of chemotherapy to radiotherapy (7–10). However, in spite of recent advances in imaging, chemotherapy administration, and radiotherapy planning and delivery, outcomes from cervical cancer have not markedly improved, even in developed countries where the most cutting-edge therapies are readily available (11).

Novel treatment strategies are urgently needed to improve outcomes from cervical cancer. One challenge to novel therapy development is that clinical trials are often conducted in high-income countries where research resources are the greatest; however, the incidence of cervical cancer is lowest. Furthermore, outcomes from clinical trials conducted in high-income countries may not be generalizable to low- and middle-income countries, which face unique challenges in cancer treatment accessibility and administration. Partnership between clinicians and researchers in developing and developed nations is vital to generating treatment paradigms with worldwide applicability and efficacy.

Cancer clinical trial access is scare in many developing countries, as the research support and infrastructure needed to enroll patients is often unavailable. The Cervix Cancer Research Network (CCRN), a subsidiary of the Gynecologic Cancer InterGroup (GCIG), is a multi-national, multi-institutional consortium of physicians and scientists focused on improving cervical cancer outcomes worldwide by making cancer clinical trials available in low-, middle-, and high-income countries. In this manuscript, we describe the early activities of the CCRN, with a focus on describing a model of collaborative capacity-building, with the overall goal of promoting cervical cancer research and improving access to novel therapies.

## Materials and Methods

### History

The CCRN is a subsidiary of the GCIG, a non-profit network of appointed representatives from international cooperative research groups for clinical trials in gynecologic cancers. The GCIG was established in 1990s with the goal of promoting and conducting high quality clinical trials to improve outcomes for women with cancers of the ovary, uterus, and cervix. The GCIG has been highly successful in completing clinical trials, publishing results, and developing consensus conferences.

The CCRN developed to address the lack of cervical cancer clinical trials, increase enrollment on existing trials, and improve the standards of cancer care in low- and middle-income countries. In light of the limited improvement in survival in locally advanced cervical cancer in the decades since chemoradiation became the standard of care, the vision of the CCRN was to provide infrastructure and support for cancer clinical trials in developing nations that have a significant burden of cervical cancer.

### Governance

The CCRN reports to and is guided by the Executive Board of Directors of the GCIG. This Board has regularly scheduled teleconferences and semi-annual meetings. The chair and co-chair of the CCRN are elected for 3-year terms by voting members. The chair of the CCRN serves on the Executive Board of the GCIG and a formal report of activities and progress is made to the membership at the General Assembly at each semi-annual meeting.

### Early development

**Figure 1:**
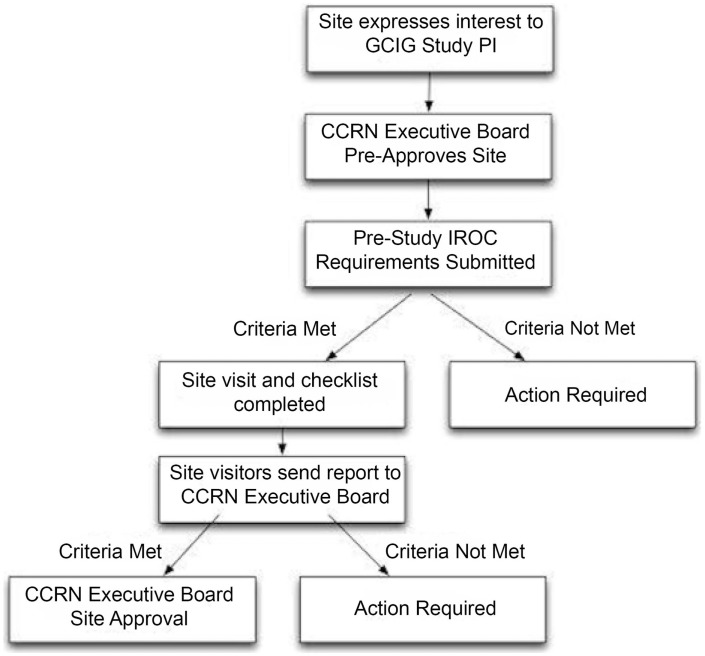
**Workflow for CCRN site approval**.

The mission of the CCRN was formulated by the committee chair and participating members. The literature was evaluated for best practices for clinical trials within gynecologic cancers, with emphasis on methods for low- and middle-income countries in which clinical trial resources are often limited. The CCRN then developed standard operating procedures (SOP) to evaluate potential participating sites to ensure appropriate infrastructure prior to clinical trial enrollment. The principal investigators of the CCRN trials normally select potential sites. The SOP workflow is demonstrated in Figure 1.

The SOP includes a pre-qualifying questionnaire to evaluate clinical activity, site resources, clinical trials infrastructure, radiation therapy treatment records, radiotherapy quality assurance, and clinical management documentation (Table 1). Additionally, participation in a radiotherapy beam measurement program is required every 2 years to determine the stability of the output of linear accelerators from each participating center. This is typically done with thermal luminescent dosimetry or more recently, optically stimulated luminescent dosimetry. With support from the National Cancer Institute of the United States, the Imaging and Radiation Oncology Core in Houston, TX, USA, has been instrumental in partnering with the CCRN to provide quality assurance checks.

**Table 1:** **Pre-qualifying questionnaire**.

Assessment category	Question
Clinical volume	• Average number of new cancer patients seen per year?
	• Average number of new gynecologic cancer patients seen per year?
	• Average number of new cervix cancer patients seen per year?
Pathology/hematology resources	Availability of the following resources on site (yes/no)
	• Routine hematology
	• Routine biochemistry
	• Routine anatomic pathology
	• Long-term specimen storage
	• Designated gynecologic pathologist
	• Specialized pathology services
	• Transfusion facility
	• Critical care facility
Radiology Resources	Availability of the following resources on site (yes/no)
	• Plane X-ray
	• Ultrasound
	• Computed Tomography (CT)
	• Positron-Emission Tomography (PET)
	• Magnetic Resonance Imaging (MRI)
	• PET/CT
	• Dedicated gynecologic radiologist
Technology support	• Email available during working hours?
	• Access to computers for doctors, technologists, data managers, and nurses?
	• Is the facility capable of digital data exchange?

For potential study sites deemed eligible after the pre-qualifying survey, a site visit must be performed by an audit team to evaluate the appropriateness and readiness to participate in CCRN trials. Infrastructure, the physical plant, and human resources are evaluated to ensure that clinical trial participation can succeed. The audit team typically includes one clinical specialist and one clinical trials manager. Various measures of quality assurance are performed, depending on the requirements of the available clinical trial. To date, the CCRN has received limited funding from the International Gynecologic Cancer Society (IGCS) and the GCIG, as well as support from the NCI.

## Results

To date, 13 different CCRN site visits have been performed. Of these 13 sites visited, 10 have been approved as CCRN sites including Tata Memorial Hospital, India; Bangalore, India; Trivandrum, India; Ramathibodi, Thailand; Siriaj, Thailand; Pramongkutklao, Thailand; Ho Chi Minh, Vietnam; Blokhin Russian Cancer Research Center; the Hertzen Moscow Cancer Research Institute; and the Russian Scientific Center of Roentgenoradiology. Approval with contingencies has been granted to sites in Cluj, Romania, and Minsk, Belarus.

Through significant efforts within the Cervix Cancer Committee at the GCIG, four multi-national cervical cancer clinical trials suitable for both developed and developing nations have successfully been opened.

1. The Tri-weekly Administration of Cisplatin in LOcally Advanced Cervical Cancer Trial (TACO Trial), developed by investigators from the Korean Gynecologic Oncology Group (KGOG) and the Thai Cooperative Group, is a randomized phase III study that compares weekly chemotherapy for advanced cervix cancer to every-3-week chemotherapy. Preliminary data from a phase II trial by the KGOG suggest that every-3-week chemotherapy may confer a survival benefit (12).
2. The OUTBACK Trial is led by investigators from the Australia/NewZealand Gynecologic Oncology Group (ANZGOG). This study is a randomized phase III trial evaluating the efficacy of extended adjuvant chemotherapy in women with advanced cervix cancer compared to the standard of weekly cisplatin chemotherapy and definitive radiotherapy. The OUTBACK chemotherapy consists of four cycles of carboplatin and paclitaxel chemotherapy administered after standard concurrent chemoradiotherapy. The rationale for the study is a meta-analysis of several studies that showed adjuvant chemotherapy to be a promising approach (13).
3. The INTERLACE Trial is headed by the National Cancer Research Institute (NCRI) from the United Kingdom. This is a randomized phase III study evaluating neoadjuvant chemotherapy prior to concurrent chemoradiotherapy for women with advanced cervix cancer compared to concurrent chemoradiotherapy alone. The goal of this study is to improve compliance with additional chemotherapy by giving it before standard chemoradiotherapy, as opposed to after standard chemoradiotherapy.
4. The SHAPE Trial is spearheaded by investigators from the NCIC Clinical Trials Group in Canada. This randomized phase III trial is evaluating radical hysterectomy versus simple hysterectomy in women with early-stage cervix cancer. The primary endpoint is freedom from pelvic failure.

Each approved CCRN site chooses to participate in one or more of the four available clinical trials. To date, 48 patients have been enrolled.

## Discussion

The greatest burden of cervical cancer is in developing countries, particularly parts of Africa, Central and South America, Eastern Europe, India, and other parts of Asia (1). The outcomes from locally advanced cancer remain suboptimal, and development and testing of novel therapies has occurred in countries with the lowest incidence of cervical cancer. The aim of the CCRN is to improve access to clinical trials, enhance clinical trial enrollment particularly in countries with high disease burden, and to produce treatment paradigms that are applicable and accessible to women worldwide.

There are many challenges in conducting multi-national clinical trials, particularly in low-resource settings (14, 15). Human resource training and research infrastructure development are necessary to ensure success; however, this may entail high costs. Rigorous quality assurance is also costly, but necessary to maintain the validity of the research question. Furthermore, open and rapid communication among study coordinators, physicians, and patients is required, but can be challenging due to language barriers and connectivity issues. Another challenge is collaboration not only with local physicians and hospitals but also with government and insurers. As an example, the CCRN sites in India have not been activated secondary to strict requirements by the Indian Government for complete trial insurance, which was too expensive to be covered by the four funded CCRN trials. Finally, extraordinary care must be taken to ensure clinical trial design and conduct is in accordance with the ethics guidelines set forth by the World Medical Association’s Declaration of Helsinki and the Council for International Organization of Medical Sciences (15).

In spite of these real and complex challenges, there are tremendous opportunities to enhance clinical trials results and improve cervical cancer outcomes through collaboration, creativity, and persistence. Rapid improvements in technology, particularly internet-based approaches, have made communication and quality assurance checks more feasible, timely, and cost-effective. Investments in research training and infrastructure development have the potential to influence not only cervical cancer clinical trial involvement but also standard care and care for other types of cancers. While sophisticated translational trials involving complex imaging and biomarker measurement will be confined to core GCIG settings, pragmatic trials that are aimed at defining worldwide standard of care, as well as trials directed at practices in low- and middle-income countries, are within the capability of the CCRN.

In summary, the CCRN has developed a methodology to evaluate potential clinical trial enrollment sites in low- and middle-income countries to make cervical cancer clinical trials available in countries with the highest burden of disease. The CCRN has successfully enrolled 10 sites in developing countries to participate in four randomized clinical trials. The primary objectives are to provide novel therapeutics to regions with the greatest need and to improve the validity and generalizability of clinical trial results by enrolling a diverse sample of patients, with the ultimate goal of improving outcomes from cervical cancer worldwide.

## Conflict of Interest Statement

The authors declare that the research was conducted in the absence of any commercial or financial relationships that could be construed as a potential conflict of interest.
